# Resistance-Trained Individuals Are Less Susceptible to Oxidative Damage after Eccentric Exercise

**DOI:** 10.1155/2018/6857190

**Published:** 2018-07-17

**Authors:** Ypatios Spanidis, Dimitrios Stagos, Christina Papanikolaou, Konstantina Karatza, Andria Theodosi, Aristidis S. Veskoukis, Chariklia K. Deli, Athanasios Poulios, Sofia D. Koulocheri, Athanasios Z. Jamurtas, Serkos A. Haroutounian, Demetrios Kouretas

**Affiliations:** ^1^Laboratory of Animal Physiology, Department of Biochemistry and Biotechnology, University of Thessaly, 41500 Viopolis, Larissa, Greece; ^2^Laboratory of Exercise Biochemistry, Exercise Physiology and Sports Nutrition (SmArT Lab), Department of Physical Education and Sport Science, University of Thessaly, 42100 Trikala, Greece; ^3^Department of Nutritional Physiology and Feeding, Faculty of Animal Science and Aquaculture, Agricultural University of Athens, Iera Odos 75, 11855 Athens, Greece

## Abstract

It has been proposed that exercise-induced oxidative stress and adaptations are dependent on training status. In this study, we examined the effects of training background on free radical generation and adaptations after eccentric exercise. Forty volunteers were divided into two groups (trained and untrained) and were asked to perform eccentric exercise. Then, their blood samples were collected pre, 24, 48, and 72 hours postexercise. Biomarkers indicating oxidative damage and the antioxidant profiles of the participants were measured in plasma and erythrocyte lysate both spectrophotometrically and chromatographically. The results revealed that the untrained group depicted more severe oxidative damage (protein carbonyls, malondialdehyde), weaker antioxidant status (reduced glutathione, static and capacity oxidation-reduction potential), and weaker radical-scavenging activity (superoxide radical scavenging and reducing power) compared to the trained participants. Our findings show that trained individuals are less susceptible to oxidative damage and suggest that generalized nutritional recommendations regarding recovery after exercise should be avoided.

## 1. Introduction

The association between physical exercise and free radical generation has been established in the literature and is attributed to diverse mechanisms [1]. Numerous studies have reported that reactive oxygen species (ROS) generation postexercise leads to severe muscle damage and oxidative stress [2]. Moreover, it has been proposed that the magnitude of ROS production is directly related to exercise intensity, resulting therefore to an excessive increase in ROS production after an intense and demanding exercise [3].

Eccentric exercise is considered to be a quite demanding exercise modality. It is characterized by an active contraction and lengthening of skeletal muscle inducing severe tissue damage characterized by decreased muscle force production, increased serum creatine kinase activity, and inflammation [4]. Thus, eccentric exercise leads to cellular disruption, loss of normal function, and soreness [5]. However, despite the fact that eccentric exercise is related to severe oxidative stress induction, significant differences in oxidation levels after eccentric exercise, as well as the presence of reductive stress among individuals, have also been observed [6]. Such diversity could be possibly attributed to the training background of each individual, since, according to the literature, ROS produced during regular exercise induce adaptations by improving antioxidant capacity, mitochondrial biogenesis, insulin sensitivity, cytoprotection, and aerobic capacity of skeletal muscle [2].

Since there was a need to find a parameter that may affect individuals' response after performing eccentric exercise, the primary purpose of the present study was to examine the effects of muscle-damaging eccentric exercise on blood redox profile and to shed light on the impact of training load on redox adaptations. Furthermore, we attempted to clarify whether training background affects changes in redox biomarkers after an arduous and demanding exercise modality, such as eccentric exercise. The idea for the present study emerged from past works of our scientific team and others that correlated oxidative damage (protein carbonyls (PC), thiobarbituric acid reactive substances (TBARS)) and the weaker redox status of individuals with muscle soreness after bouts of eccentric exercise. In addition, the diversity among participants is highlighted [6–9].

It is evident that this approach will try to fill the gaps in literature regarding the response of trained and untrained people after performing eccentric exercise and examine whether an individual or group approach should be conducted. This would also help to improve the response of exercising individuals regarding recovery and health status after specific interventions (e.g., nutrition or administration of antioxidant supplements). We hypothesized that untrained people may display a compromised antioxidant profile and, therefore, will be more susceptible to oxidative and muscle damage compared to the trained ones as they are less adapted in performing bouts of exercise.

## 2. Materials and Methods

### 2.1. Subjects

Twenty-four male and sixteen female volunteers (age 22.5 ± 0.58 years, height 175.1 ± 1.6 cm, and weight 75.4 ± 2.3 kg) participated in the present study. The selection of the participants was based on their athletic (i.e., training) background. In order to cluster the participants into two groups, they were asked to provide details about their athletic background. According to them, the trained group consisted of 15 males and 7 females. Seventeen participants regularly performed resistance exercise (i.e., weightlifting) recreationally at least 4 times per week, while the remaining 5 mostly performed a combination of aerobic and resistance exercises. The untrained group comprised 18 participants (9 males and 9 females), who had never been in contact with any kind of exercise.

Subjects have not suffered any musculoskeletal injuries to the lower limbs that would limit their ability to perform the exercise protocol. Additionally, the participants were asked to abstain from smoking and from consuming alcohol and nutritional supplements, as well as from engaging in any kind of exercise for over a week before the study and during the experiment. However, there were no limitations regarding food intake before or during blood sampling. Body mass was measured to the nearest 0.5 kg (BeamBalance 710, Seca, United Kingdom), while the subjects were lightly dressed and barefoot. Standing height was measured to the nearest 0.5 cm (Stadiometer 208, Seca). A written informed consent to participate in the study was provided to and was signed by all participants after they had been informed of all benefits, risks, and discomforts of the investigation.

### 2.2. Study Design

The participants of the present study were divided into two groups (i.e., trained and untrained) according to their athletic background, as mentioned in Section 2.1. Blood samples were collected before and 24 h, 48 h, and 72 h after performing the eccentric exercise protocol described in the next paragraph. Plasma and erythrocyte lysate samples were isolated after blood collection and stored at −80°C until the biochemical analyses were performed.

### 2.3. Eccentric Exercise Protocol

An eccentric exercise session was performed on an isokinetic dynamometer (Cybex Norm, Ronkonkoma, NY) and exercise protocols were undertaken from the seated position (120° hip angle) with the lateral femoral condyle aligned with the axis of rotation of the dynamometer. Participants were coupled to the dynamometer by an ankle cuff, attached proximal to the lateral malleolus, and finally stabilized according to the manufacturer's instructions. Participants completed 5 sets of 15 eccentric maximal voluntary contractions (knee range, 0° full extension to 90° flexion) at an angular velocity of 60°/s. A 2 min rest interval was used between sets and the total workout time was 15 min. Before the exercise session, subjects performed a 10 min warmup consisting of cycling on a Monark cycle ergometer (Vansbro, Sweden) at 70–80 rpm and 50 W.

### 2.4. Blood Sample Preparation

The blood samples were drawn from a forearm vein in ethylenediaminetetraacetic acid (EDTA) and heparin tubes at four different time points, namely, before exercise and 24, 48, and 72 h postexercise. Subsequently, they were centrifuged (1370*g*, 10 min, and 4°C) and the supernatant (i.e., plasma) was collected. The remaining packed erythrocytes were lysed with 1 : 1 (*v*/*v*) distilled water (dH_2_O), inverted vigorously, and centrifuged (4020*g*, 15 min, and 4°C). The supernatant, which is the erythrocyte lysate, was then collected. The plasma and erythrocyte lysate samples were then stored at −80°C until further biochemical analysis.

### 2.5. Biochemical Analyses

PC were determined in plasma as described in a previous work of our team [10]. Briefly, 50 *μ*l of 20% trichloroacetic acid (TCA) was added to 50 *μ*l of plasma, and this mixture was incubated in an ice bath for 15 min and centrifuged (15,000*g*, 5 min, and 4°C). The supernatant was discarded and 500 *μ*l of 10 mM 2,4-dinitrophenylhydrazine (in 2.5 N HCl) for the sample, or 500 *μ*l of 2.5 N HCl for the blank, was added to the pellet. The samples were incubated in the dark at room temperature (RT) for 1 h with intermittent vortexing every 15 min and centrifuged (15,000*g*, 5 min, and 4°C). The supernatant was discarded, and 1 ml of 10% TCA was added. Then, the samples were vortexed and centrifuged (15,000*g*, 5 min, and 4°C). The supernatant was discarded, and 1 ml of ethanol-ethyl acetate mixture (1 : 1 *v*/*v*) was added. Then, the samples were vortexed and centrifuged (15,000*g*, 5 min, and 4°C). This step was repeated twice. The supernatant was discarded, and 1 ml of 5 M urea (pH = 2.3) was added. Then, the samples were vortexed and incubated at 37°C for 15 min. The samples were then centrifuged (15,000*g*, 3 min, and 4°C), and the absorbance was monitored at 375 nm. Total plasma protein was determined using Bradford's method via a standard curve of solutions with known bovine serum albumin concentrations.

 For plasma malondialdehyde (MDA) assessment as a biomarker of lipid peroxidation, one spectrophotometric (TBARS) and one chromatographic (high-performance liquid chromatography with diode-array detector, HPLC-DAD) method was applied. For TBARS determination, 100 *μ*l of plasma was mixed with 500 *μ*l of 35% TCA and 500 *μ*l of Tris-HCl (200 mM, pH = 7.4) and incubated for 10 min at RT. A total of 1 ml of 2 M sodium sulfate (Na_2_SO_4_) and 55 mM thiobarbituric acid (TBA) (2.84 g of Na_2_SO_4_ and 0.08 g of TBA diluted in 10 ml of dH_2_O) was added and the samples were incubated at 95°C for 45 min. The samples were cooled on ice for 5 min, vortexed after adding 1 ml of 70% TCA, and centrifuged (15,000*g*, 3 min, and 20°C). Then, the absorbance of the supernatant was monitored at 530 nm. TBARS concentration was calculated on the basis of the molar extinction coefficient of MDA [10].

Regarding plasma MDA determination by chromatography (HPLC-DAD), a method described by Spirlandeli et al., [11] was used. Briefly, 100 *μ*l of plasma was added to 700 *μ*l of 1% phosphoric acid and 200 *μ*l of 42 mM TBA. The mixture was vortexed and heated for 40 min in a water bath at 100°C. Afterwards, 250 *μ*l of the mixture was added to 250 *μ*l of 1 M sodium hydroxide in methanol (1 : 6), centrifuged (10,000*g*, 5 min, and 20°C), filtered through a Milli-RO 10 Plus and a Milli-Q Plus plant (final pore size 0.2 mm; Millipore, Bedford, MA). Then, 20 *μ*l of the supernatant was injected in the HPLC apparatus at a flow rate of 1 ml/min. The absorbance was monitored at 532 nm. For the MDA standard curve, a stock solution of 100 *μ*M MDA was prepared in 0.01 mM HCl. Dilutions from stock MDA solutions of 2–14 *μ*M were then performed. The same treatment described for plasma was used for the standard. The method was carried out by using a 5 *μ*m C18 reverse-phase column (4.6 mm × 250 mm) in a Hewlett Packard HP1100 Series HPLC Value System (Agilent Technologies, Waldbronn, Germany) equipped with a quaternary pump, autosampler, degasser, and diode array detector (DAD). Retrieval and processing of chromatographic data was performed with Chemstation Software.

In total antioxidant capacity (TAC) determination, 20 *μ*l of plasma was added to 480 *μ*l of 10 mM sodium potassium phosphate (pH = 7.4) and 500 *μ*l of 0.1 mM 2,2-diphenyl-1-picrylhydrazyl (DPPH^∙^). The samples were incubated in the dark for 30 min at RT and centrifuged (20,000*g*, 3 min, and 20°C). Then, the absorbance was monitored at 520 nm [10]. In GSH, 20 *μ*l of erythrocyte lysate treated with 5% TCA was mixed with 660 *μ*l of 67 mM sodium potassium phosphate (pH = 8.0) and 330 *μ*l of 1 mM 5,5-dithiobis (2 nitrobenzoic acid) (DTNB). The samples were incubated in the dark at RT for 45 min and the absorbance was monitored at 412 nm. GSH concentration was calculated relative to a calibration curve made using commercial standards [10].

Catalase (CAT) activity in the erythrocyte lysate was measured as previously described [10]. Specifically, 4 *μ*l οf erythrocyte lysate (diluted 1 : 10) was added to 2991 *μ*l οf 67 mM sodium potassium phosphate (pH = 7.4) and the samples were incubated at 37°C for 10 min. Five microliters of 30% hydrogen peroxide (H_2_O_2_) was added to the samples and the absorbance was monitored at 240 nm for 130 sec. CAT activity was calculated on the basis of the molar extinction coefficient of H_2_O_2_.

The superoxide anion radical-scavenging ability of plasma was measured using a slightly modified protocol of Ak and Gülçin, [12]. In this method, superoxide anion (O_2_^∙−^) is generated in a phenazine methosulfate and reduced nicotinamide adenine dinucleotide (PMS-NADH) system by NADH oxidation and it reduces the yellow dye of nitroblue tetrazolium (NBT^2+^) to the blue colored formazan. More specifically, 125 *μ*l of 300 *μ*M NBT^2+^, 125 *μ*l of 468 *μ*M NADH, and 50 *μ*l of plasma were added into 625 *μ*l of 16 mM Tris-HCl (pH = 8.0). The reaction is initiated by the addition of 125 *μ*l of 60 *μ*M PMS to the mixture. The samples were incubated for 5 min and the absorbance was monitored at 560 nm. Plasma antioxidants are acting as inhibitors to the blue colored formazan formation. The O_2_^∙−^ radical-scavenging activity was calculated according to (1):

(1) $$\begin{array}{c} \%\;Superoxide\;radical\;scavenging\;activity=\left[\frac{\left({Abs}_{control}-{Abs}_{sample}\right)}{{Abs}_{control}}\right]\times 100, \end{array}$$

where Abs_control_ and Abs_sample_ are the absorbance values of the control and the tested sample, respectively.

In the reducing power assay, a plasma sample was dissolved in phosphate buffer (0.2 M, pH = 6.6) at different concentrations. An aliquot (250 *μ*l) of the sample solution was added to 250 *μ*l of 1% potassium ferricyanide and incubated at 50°C for 20 min. The samples were cooled on ice for 5 min. Then, 250 *μ*l of 10% TCA was added and the samples were centrifuged (1700*g*, 10 min, and 25°C). Subsequently, 250 *μ*l of dH_2_O and 50 *μ*l of 0.1% ferric chloride were added to the supernatant and the samples were incubated at RT for 10 min. The absorbance was monitored at 700 nm [13].

Regarding the assay for hydroxyl radical- (OH^∙^-) scavenging activity, 75 *μ*l of plasma dissolved in dH_2_O at different concentrations was added to 450 *μ*l of 0.2 M sodium phosphate buffer (pH = 7.4), 150 *μ*l of 10 mM 2-deoxyribose, 150 *μ*l of 10 mM FeSO_4_-EDTA, 525 *μ*l of dH_2_O, and 150 *μ*l of 10 mM H_2_O_2_. Then, the samples were incubated at 37°C for 4 h. Afterwards, 750 *μ*l of 2.8% TCA and 750 *μ*l of 1% TBA were added, and the samples were incubated at 95°C for 10 min. Then, the samples were cooled on ice for 5 min, centrifuged (1700*g*, 10 min, and 25°C), and the absorbance was monitored at 520 nm. In each experiment, the sample without H_2_O_2_ was considered as blank and the sample without protein as control. The OH^∙^ scavenging activity was calculated according to (2):

(2) $$\begin{array}{c} \%Hydroxyl\;radical\;scavenging\;activity=\left[\frac{\left({Abs}_{control}-{Abs}_{sample}\right)}{{Abs}_{control}}\right]\times 100, \end{array}$$

where Abs_control_ and Abs_sample_ are the absorbance values of the control and the tested sample, respectively [14].

Oxidation-reduction potential (ORP) in plasma was determined by a novel method using the RedoxSYS Diagnostic System (Luoxis Diagnostics Inc., Englewood, CO, USA). Specifically, ORP is an integrated measure of the balance between the pool of oxidants (e.g., oxidized thiols, O_2_^∙−^, OH^∙^, H_2_O_2_, NO^∙^, ONOO^∙^, and transition metal ions) and the pool of reductants (e.g., free thiols, ascorbate, *α*-tocopherol, *β*-carotene, and uric acid). It has been shown that this is an effective, fast, and accurate method for the determination of oxidative stress induced by an ultramarathon mountain race, eccentric exercise, and a strenuous basketball season [6, 10, 15]. The system consists of a battery-powered reader and small sensors that require limited sample manipulation, as it measures ORP within 4 min in 20 *μ*l of heparinized mammalian plasma samples. Static oxidation-reduction potential (sORP) value displays the integrated balance of oxidants and reductants in a sample and is expressed in millivolts (mV). Capacity oxidation-reduction potential (cORP) is the amount of the antioxidant pool in the human body and is expressed in microcoulombs (*μ*C). High sORP values and low cORP values indicate the presence of oxidative stress [10].

### 2.6. Assessment of Delayed Onset Muscle Soreness (DOMS)

Perceived soreness of the participants as a measure of DOMS was rated by them on a scale ranging from 1 (i.e., no pain), 5 (i.e., moderate pain), to 10 (i.e., very strong pain) during walking (DOMSw) and the squat movement (DOMSsq).

### 2.7. Statistical Analysis

The distribution of the biomarker values in each sample was examined by the Shapiro-Wilk test and was found not to differ significantly from normality. Data were analyzed using two-way ANOVA followed by Dunnett's test for multiple pairwise comparisons. Correlations between oxidative stress biomarkers were examined by Spearman's correlation analysis. The level of significance was set at *p* < 0.05. Data are presented as mean ± SEM. For all statistical analyses, SPSS version 20.0 (SPSS, Inc., Chicago, IL, USA) was used.

## 3. Results

### 3.1. Muscle Soreness

Eccentric exercise resulted in significant increases in DOMS levels of the trained group ranging between 3.49- and 4.52-fold during walking and between 3.58- and 4.33-fold after squatting. The corresponding data collected by the untrained group after walking and the squat movement increased significantly from 3.44- to 5.34-fold and from 3.44- to 5.00-fold, respectively (Table 1). Moreover, the correlation between DOMS levels and oxidative stress markers exhibited significant negative correlations between DOMS, cORP, and reducing power in the untrained group. Specifically, there was a negative correlation between DOMS squat and cORP 48 h postexercise while negative correlations were also obtained between DOMS walking, cORP, and reducing power at the 72 h postexercise time point (Table 2).

### 3.2. Oxidative Stress Biomarkers

#### 3.2.1. Protein Oxidation

Protein carbonyl levels in the untrained group were significantly increased 48 h postexercise by 14.67% compared with the preexercise value and were also significantly higher compared with the corresponding results of the trained group (Table 3). On the contrary, protein carbonylation of the trained group was slightly, but not significantly, decreased at all time points (Table 3).

#### 3.2.2. Lipid Peroxidation Measured by Spectrophotometry

TBARS levels were increased in the trained group by 10.25%, 8.50%, and 14.98% at 24, 48, and 72 h, respectively, while the corresponding results in the untrained individuals revealed significant increases by 18.29%, 26.89%, and 13.49%, respectively (Table 3). The TBARS levels of the untrained group were significantly higher compared to the trained group 48 h postexercise (Table 3).

#### 3.2.3. Lipid Peroxidation Measured by Chromatography

**Figure 1:**
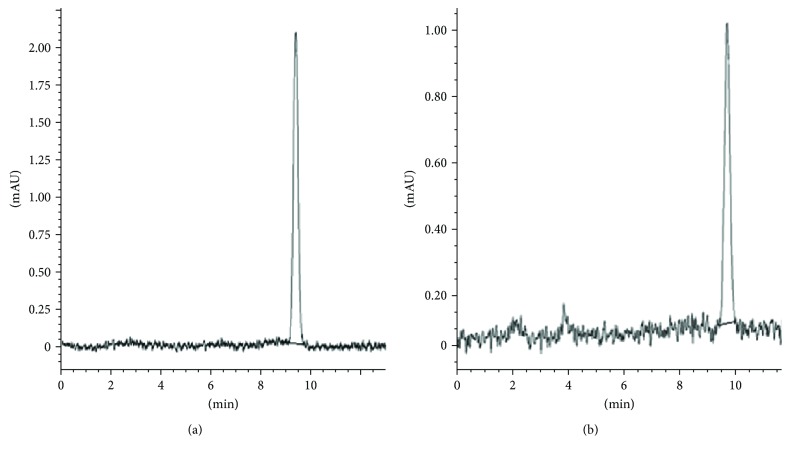
Representative chromatograms of plasma spiked with 12 *μ*M MDA (a) and a plasma sample of a volunteer containing 3.79 *μ*M MDA (b).

*(1) Validation (Linearity, Precision, and Recovery)*. After choosing the pretreatment procedure and establishing the chromatographic conditions for the analysis, the method was validated. Firstly, a pooled human plasma sample, spiked with 2, 4, 6, 8, 10, and 12 *μ*Μ of MDA, and a calibration curve was obtained. A similar procedure was also followed for the determination of the aqueous curve by using the same concentrations of MDA. Linearity and reproducibility were evaluated by linear regression. The equations obtained by the least squared regression were *y* = 3.863*x* + 11.651 for plasma curves and *y* = 4.4151*x* − 4.1043 for aqueous curves, and the values to *R*^2^ were 0.9952 and 0.9982 for plasma and aqueous curves, respectively. Moreover analytical curves (i.e., peak area of each concentration from spiked plasma against area from aqueous MDA standards) exhibited an excellent linearity having a correlation coefficient more than 0.995. Τhe relative recovery for the MDA-TBA complex was assessed at three concentrations of 0.5, 1.0, and 1.5 *μ*M, and the average recovery was counted at 98.06% (ranged from 97.54% to 98.58%). The intra- and interday precision of this specific proposed method was determined by counting standard spiked plasma solutions. Specifically, regarding the interday precision, three different spiked plasma solutions (0.5, 1.0, and 1.5 *μ*Μ MDA) were analyzed in triplicate for 5 consecutive days each. Intraday precision was counted by measuring a plasma solution spiked with 1.7 *μ*Μ of MDA plasma sample for seven times within the same day. The precisions were expressed in % RSD and calculated at 0.43% and 0.31% for the inter- and intraday tests, respectively, which are in the range of acceptability and accuracy [16]. It is worth mentioning that the average retention time was at 9.55 min (Figure 1).

*(2) Malondialdehyde Levels*. As far as the chromatographic determination of MDA levels is concerned, the results were similar in both trained (i.e., MDA levels increased at all time points by 14.07%, 14.70%, and 21.79%) and untrained groups (i.e., MDA levels increased at all time points by 15.83%, 14.55%, and 23.65%) (Table 3).

**Figure 2:**
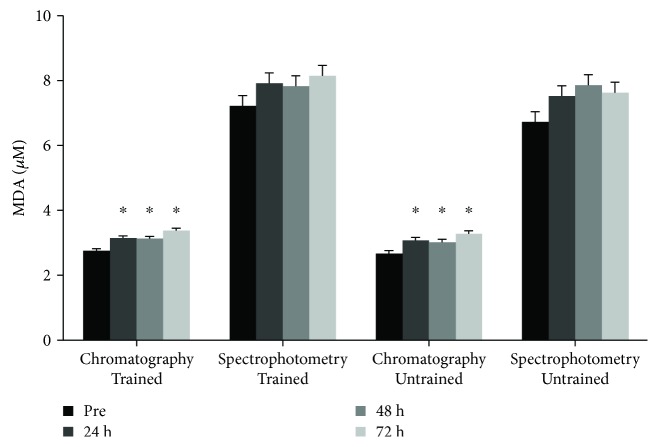
Comparison of malondialdehyde (MDA) concentrations measured chromatographically (HPLC) and spectrophotometrically (TBARS). ^∗^Statistically significant compared with preexercise value.

**Figure 3:**
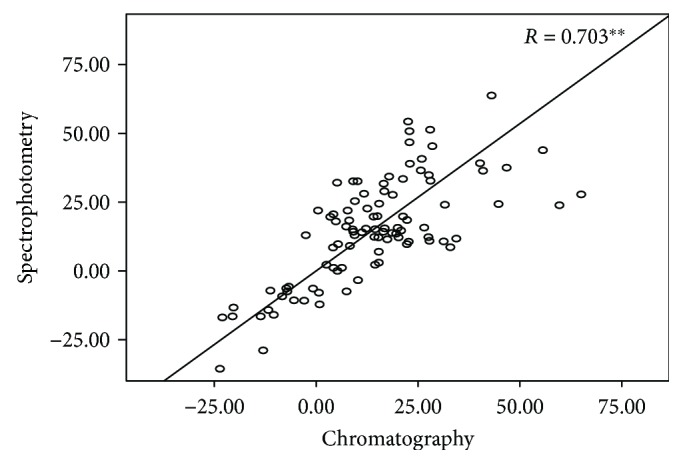
Spearman's correlation coefficient (R) and solid line for percentage (%) alterations of MDA concentrations measured chromatographically (HPLC) and spectrophotometrically (TBARS). ^∗∗^Significant correlation (*p* < 0.01).

*(3) Comparison between the MDA Concentrations Measured by Spectrophotometry and Chromatography.* All tested groups exhibited a 2.5-fold increase in MDA concentration measured by spectrophotometry (ranged from 5.5 to 9.2 μM) compared with chromatography (ranged from 2.2 to 4.7 μM, resp.) (Figure 2). The percentage alterations of MDA concentrations postexercise compared to preexercise were also similar between the two methods (Table 3). This similarity was also confirmed by the significant correlation between the percentage alterations of MDA values obtained by the two analytical techniques (*r* = 0.703, *p* < 0.01) (Figure 3).

#### 3.2.4. GSH Levels

As depicted in Table 3, GSH was significantly increased in the trained group 24 and 48 h postexercise by 12.63% and 23.09%, respectively. Furthermore, there was also a significant increase in the trained group compared with the untrained participants 48 h postexercise. On the contrary, GSH levels were significantly decreased in the untrained group by 9.64% 72 h postexercise compared to preexercise (Table 3).

#### 3.2.5. Catalase Activity

Regarding catalase activity, no significant effects were observed (Table 3).

#### 3.2.6. ORP Markers

The results from ORP marker analysis as estimated by the RedoxSYS system showed significant differences between the two groups at all time points (Table 3). In particular, the untrained group displayed significant increases at all time points postexercise by 4.65%, 4.01%, and 7.45%, respectively, compared to preexercise, indicating induced oxidative status (Table 3). The cORP analysis indicated induced reductive status postexercise compared to preexercise in the trained group (Table 3). In fact, cORP levels were significantly increased by 30.57% and 27.15% 24 and 48 h postexercise, respectively, compared with preexercise values (Table 3).

#### 3.2.7. Determination of Antioxidant and Free Radical Scavenging Capacity

Regarding TAC, no significant differences were observed either between pre- and postexercise or between trained and untrained groups (Table 3). In the reducing power assay, there was only a significant increase 72 h postexercise in the trained group compared to the untrained individuals (Table 3). Regarding superoxide radical-scavenging activity in the trained group, an increase 24 and 48 h postexercise by 12.16% and 7.45%, respectively, compared to preexercise was displayed (Table 3). The participants of the trained group also exhibited increased superoxide radical-scavenging capacity compared with the untrained group 24 and 48 h postexercise (Table 3). However, no significant alteration was observed in the hydroxyl radical-scavenging capacity either between pre- and postexercise or between trained and untrained groups (Table 3).

Moreover, a Spearman correlation analysis was conducted for examining the possibility of a potential correlation between the above four biomarkers (Table 4). No significant correlations were observed in any of the tested groups, apart from a moderate significant correlation (*R* = 0.514) between superoxide radical-scavenging capacity and reducing power levels in the trained group.

## 4. Discussion

In the present study, the effects of eccentric exercise on oxidative stress and inflammation between trained and untrained individuals were examined. A first approach in the evaluation of exercise-induced muscle damage was the assessment of muscle pain, as eccentric exercise has been shown to cause delayed onset muscle soreness (DOMS) [6]. DOMS has been considered the major cause of reduced exercise performance, impaired muscle strength, and psychological discomfort for both trained and untrained humans [17]. In the present study, DOMS levels in the trained group were lower than those in the untrained group. It is also worth mentioning that the peak muscle soreness was detected 48 h postexercise in both groups, being in accordance with other studies [18]. Eccentric exercise-induced inflammation may be the main cause of ROS production after a bout of exercise as it leads to migration of phagocytic cells to the damaged tissue. The forthcoming respiratory burst results in the production of ROS, such as superoxide and hydroxyl radicals [6]. Statistical analysis confirmed the aforementioned hypothesis, as DOMS in the untrained group displayed a significant negative correlation with cORP and reducing power, which depict the antioxidants' reserves and scavenging activity, respectively. However, a “preconditioning” of muscle during exercise may reduce susceptibility to inflammation response after performing a new bout of eccentric exercise, and consequently the inflammatory response is not so extended, resulting in weaker muscle pain. The latter confirms the notion regarding the relationship between inflammatory and pain response and may account for the difference in muscle pain between the two examined groups of our study [19].

As regard to protein carbonylation, it typically occurs several hours after eccentric exercise and generates a substantial amount of ROS via multiple mechanisms [20]. Our results indicated a more effective protection of proteins from oxidation (i.e., reduction of PC concentration) in the trained group compared to the untrained individuals postexercise assuring the impact of training background [19]. Interestingly, another study has shown an increase in protein oxidation levels of “nonresistance” trained women after eccentric exercise [20]. Considering that in this experiment the majority of trained individuals (17/22) were accustomed to resistance training, it seems that the type of exercise may affect the range of muscle injury and the forthcoming protein carbonylation.

Lipid peroxidation did not show any significant alterations between the tested groups when measured spectrophotometrically using the TBARS assay. However, increased MDA concentrations were observed in both groups at all time points compared with preexercise samples. This is usually observed several hours to days after acute resistance exercise probably triggered by leukocyte and macrophage infiltration and/or xanthine oxidase activation due to the ischemia-reperfusion process [21]. Since the TBARS assay has received criticism due to a lack of specificity that increases the noise of the measurements [22, 23], we also performed HPLC-DAD, a more specific method for MDA determination in plasma, in order to compare these two methods. The results indicated an approximately 2.5-fold increase in the absolute values of MDA concentration measured spectrophotometrically compared with the chromatographic results in both groups. This finding confirmed that MDA is determined in a more sensitive manner by HPLC than by spectrophotometry, since in TBARS assay, TBA reacts apart from MDA with other compounds such as sugars, amino acids, and aldehydes [11]. Interestingly, a similar difference between the two performed assays has also been previously reported [11, 23]. However, the TBARS assay exhibited a good matching with chromatography regarding the postexercise percentage alterations of MDA concentrations, a fact that was also verified by the significant correlation between the two assays (Figure 3). Therefore, regardless of the obtained overestimated absolute values, alterations of MDA concentration can be reliably described using spectrophotometry (i.e., TBARS). Thus, (3) can be used by researchers for the accurate spectrophotometric calculation of MDA concentration:

(3) $$\begin{array}{c} Chromatographic\;\left[MDA\right]\;\left(\mu M\right)=0.4\;spectrophotometric\;\left[MDA\right]\;\left(\mu M\right). \end{array}$$

Regarding GSH levels, they increased in the trained compared to the untrained individuals at all tested time points and especially 48 h postexercise. In that sense, it has been proposed that regular exercise induces adaptations due to the repeated activation of antioxidant genes and proteins leading to a higher antioxidant capacity and, therefore, to a more effective neutralization of ROS [2]. GSH-related antioxidant enzymes play a significant role in GSH synthesis and regeneration and, therefore, this explains the GSH increase in trained participants [24].

Similarly, ORP markers also indicated that oxidative stress was lower in the trained individuals. Specifically, sORP levels in the untrained group were found not only significantly higher postexercise compared with preexercise values but also elevated compared to the trained participants suggesting oxidative stress induction. Our group has previously reported that this marker was increased and associated with oxidative stress induction after endurance and strenuous exercise [6, 10]. The obtained result of the cORP assay, which indicates antioxidant capacity, was also in accordance with sORP, as it was higher in the trained participants. Thus, it becomes evident that untrained individuals are more vulnerable to ROS generation and inflammation response after muscle injury. The improved capability of trained people to decrease ROS levels imply that they are better protected from exercise-induced oxidative damage.

The increased antioxidant capacity of the trained participants was also confirmed by the significant increase in O_2_^∙−^ scavenging capacity postexercise compared to preexercise as well as compared to their untrained counterparts. According to the literature, SOD plays a predominant role in scavenging O_2_^∙−^ in plasma [25]. Interestingly, studies have shown that trained individuals exhibited high SOD levels, thus coping with O_2_^∙−^ more effectively [26]. Similarly, reduction of Fe(III) to Fe(II) as determined by the reducing power assay was higher in the trained in comparison with the untrained individuals. It is apparent that the concentrations of plasma antioxidant molecules, such as uric acid, *α*-tocopherol, bilirubin, and ascorbic acid are elevated in individuals with an athletic background [27]. The high GSH levels in the trained group may account for their higher reducing capacity, since GSH acts as an antioxidant by donating hydrogen atoms in the regeneration of the antioxidant vitamins E and C [28]. On the other hand, no significant alterations were observed in OH^∙^ scavenging levels after exercise compared with preexercise samples, whereas trained individuals were again more efficient in neutralizing OH^∙^ 72 h postexercise compared to untrained individuals.

Finally, the lack of significant correlations between the examined biomarkers is worth mentioning (apart from RP and OH^∙^ in the trained group at 72 h). This fact confirms the notion that oxidative stress induction and the following adaptations based on the activation of the antioxidant mechanisms is a complex process depending on various physiological, biochemical, and genetic factors that vary considerably between individuals [29]. This particular conclusion is very important as it suggests a personalized approach for counteracting eccentric exercise-induced oxidative stress. Undoubtedly, it suggests that a specific formulation of each person's diet, according to his oxidative status and based on supplementation with the appropriate antioxidants days after performing bouts of exercise, may lead to a faster and more efficient recovery. Regarding the present study, the nutritional intake of the participants was not thoroughly examined. However, according to their report, they did not consume higher amounts of proteins through their normal diet. Furthermore, we suggested that they should abstain from any unusual nutritional as well as antioxidant supplementation a week before, until the end of the experiment. Therefore, we believe that their nutrition did not affect our results. Nevertheless, supplementation is considered a double-edge sword, as it should only be applied in a severe oxidative stress condition after strenuous exercise. Otherwise, it can interfere with muscle adaptations and damaged-tissue regeneration [30, 31].

Taking the above data into consideration, it is clear that eccentric exercise induced reductive stress or no stress instead of oxidative stress in trained individuals, contrary to what is expected after such a demanding exercise bout. Indeed, reductive stress has also been observed by our research group in athletes who participated in top level basketball competitions as well as in individuals who have undergone a 103 km ultramarathon mountain race [10, 15]. Nikolaidis et al. [19] have reported that a repeated bout of lengthening contractions induced much less muscle damage and blood exercise oxidative stress than the first bout, a key information in our effort to analyze our findings. It seems therefore that trained individuals regularly performing eccentric contractions have performed muscle adaptations limiting in that way the exercise-induced inflammation and the subsequent free radical production generated by neutrophil and macrophage infiltration to the injury point [32].

In general, regularly performed exercise may lead to well-described adaptations of the cardiovascular and muscular system. Important responses at the intramyocellular level include increases in size and number of mitochondria as well as induction of the antioxidant enzyme activities [33, 34]. It has been proposed that exercise causes an activation of mitogen-activated protein kinases (MAPKs: p38, ERK 1, and ERK 2) that subsequently activates nuclear factor *κ*B (NF-*κ*B) in rat gastrocnemius muscle and consequently the expression of important enzymes associated with defense against ROS (i.e., Mn-SOD and Cu, Zn-SOD, CAT, and GPX1) and adaptation to exercise [35, 36]. For example, GSH-related antioxidant enzymes including glutathione reductase (GR) and GSH synthetase are also such products of the above factors' activation [24], explaining abundantly the increase of GSH in the trained participants of our study. Similarly, the activation of the mechanisms referred above, as a result of the frequent exercise, may tone up the antioxidant status of the regularly trained individuals and therefore lead to a better protection against oxidative damage and an enhanced scavenging activity against free radicals The hypothesis regarding exercise-induced responses of the trained individuals, also relies on a study, which reported that regular exercise appears to gradually increase the adaptation levels by the repeated activation of antioxidant proteins and genes [37]. However, increased free radical production may be desired or even required for normal muscle function and/or muscle regeneration [32]. Free radicals generated by neutrophils and macrophages are crucial for removing muscle tissue that has been damaged after eccentric exercise. Furthermore, they are also important as they act as signaling molecules to regulate muscle cell growth, differentiation, and proliferation in the context of damaged tissue repair [38].

## 5. Conclusion

Previous studies of our group with respect to the individualized monitoring of exercise-induced oxidative stress have suggested that each individual is a unique biological entity and that generalized recommendations concerning recovery after exercise should be avoided. Supporting this notion, the present study demonstrated that the training background is an important factor with high impact on eccentric exercise-induced oxidative stress and the subsequent adaptations. We expect that our findings will help the endeavor to identify the ideal approach in terms of type, duration, and intensity of conducted exercise, in conjunction to the training background of an individual and may help to better understand the phenomenon of oxidative or reductive stress after exercise. Moreover, as suggested by our work, individualized nutritional approach could help to fine-tune the recovery process and consequently improve health status and performance after eccentric exercise.

## Figures and Tables

**Table 1:** Delayed onset muscle soreness (DOMS) pre- and postexercise.

	Pre	Immediately after	24 h	48 h	72 h
*Trained*					
DOMSw	1.00 ± 0.00	4.49 ± 0.67^∗^	3.52 ± 0.48^∗^	4.52 ± 0.48^∗^	3.00 ± 0.46^∗^
DOMSsq	1.00 ± 0.00	3.58 ± 0.37^∗^	4.08 ± 0.51^∗^	4.33 ± 0.48^∗^	3.17 ± 0.51^∗^
*Untrained*					
DOMSw	1.00 ± 0.00	3.44 ± 0.38^∗^	4.21 ± 0.44^∗^	5.34 ± 0.53^∗^	4.43 ± 0.33^∗^
DOMSsq	1.00 ± 0.00	3.44 ± 0.50^∗^	4.84 ± 0.44^∗^	5.71 ± 0.55^∗^	5.05 ± 0.53^∗^

DOMSw: DOMS assessed during walking; DOMSsq: DOMS assessed after performing a squat movement. Values are expressed as mean ± SEM. ^∗^Statistically significant compared with preexercise values (*p* < 0.05).

**Table 2:** Statistical correlation between DOMS and the examined oxidative stress biomarkers 24 h, 48 h, and 72 h postexercise in both trained and untrained groups.

	PC	TBARS	TAC	GSH	CAT	sORP	cORP	SRS	RP	HRS	MDA
*Trained*											
24 hours											
DOMS sq.	0.074	0.423	0.141	0.182	0.164	0.191	−0.268	−0.154	0.349	0.336	0.390
DOMS w.	0.093	0.173	0.202	0.122	0.174	0.332	−0.252	−0.136	.301	−0.50	0.202
48 hours											
DOMS sq.	−0.272	−0.016	0.311	0.356	0.012	−0.216	0.060	−0.066	0.011	0.104	0.059
DOMS w.	0.027	0.061	0.221	0.217	0.001	−0.284	0.045	0.023	0.151	0.050	0.064
72 hours											
DOMS sq.	−0.286	−0.334	0.120	0.284	0.380	−0.057	0.136	0.369	0.084	0.108	−0.28
DOMS w.	−0.039	−0.372	0.039	0.078	0.389	−0.262	0.410	0.187	0.198	0.132	−0.25

*Untrained*											
24 hours											
DOMS sq.	−0.120	0.376	−0.357	0.156	0.029	0.075	−0.234	−0.018	−0.041	−0.13	0.193
DOMS w.	−0.168	0.164	−0.326	0.085	−0.05	−0.085	−0.170	−0.003	0.085	−0.03	0.123
48 hours											
DOMS sq.	−0.202	0.296	0.070	0.285	−0.306	−0.085	−0.463^∗^	0.176	−0.084	0.301	0.252
DOMS w.	−0.050	0.368	−0.281	0.227	−0.352	−0.308	−0.330	0.350	0.053	0.087	0.312
72 hours											
DOMS sq.	−0.269	−0.194	−0.118	0.146	−0.003	−0.188	−0.164	0.243	0.273	0.244	−0.12
DOMS w.	0.130	0.130	0.072	0.134	0.216	0.018	−0.484^∗^	0.078	−0.440^∗^	0.098	0.132

DOMSw: DOMS assessed during walking; DOMSsq: DOMS assessed after performing a squat movement; PC: protein carbonyls; TBARS: thiobarbituric acid reactive substances (malondialdehyde measured spectrophotometrically); TAC: total antioxidant capacity; GSH: reduced glutathione; CAT: catalase; sORP: static oxidation-reduction potential; cORP: capacity oxidation-reduction potential; SRS: superoxide radical scavenging; HRS: hydroxyl radical scavenging; RP: reducing power; MDA: malondialdehyde (measured by HPLC-DAD). ^∗^Statistically significant correlation (*p* < 0.05).

**Table 3:** Percentage (%) alterations of the oxidative stress biomarkers postexercise compared to baseline.

	Trained			Untrained		
	24 h	48 h	72 h	24 h	48 h	72 h
PC	−0.93 ± 3.51	−7.45 ± 4.21^#^	−8.04 ± 3.80	8.64 ± 4.27	14.67 ± 3.16^∗^	2.32 ± 3.16
TBARS	10.25 ± 2.93^∗^	8.50 ± 2.85^∗#^	14.98 ± 5.92^∗^	18.29 ± 7.86^∗^	26.89 ± 6.48^∗^	13.49 ± 6.98^∗^
TAC	1.59 ± 1.56	0.04 ± 1.51	0.91 ± 1.92	1.14 ± 1.38	1.50 ± 1.52	0.67 ± 1.20
GSH	12.63 ± 5.44^∗^	23.09 ± 7.17^∗#^	3.83 ± 3.19	−2.98 ± 3.91	1.11 ± 5.47	−9.64 ± 4.75^∗^
CAT	1.82 ± 3.68	10.22 ± 4.05	4.13 ± 4.14	6.09 ± 4.86	5.75 ± 4.58	2.58 ± 5.28
sORP	−2.11 ± 0.73^#^	−3.31 ± 1.11^#^	−0.64 ± 1.14^#^	4.65 ± 1.43^∗^	4.01 ± 1.61^∗^	7.45 ± 1.45^∗^
cORP	30.57 ± 7.02^∗#^	27.15 ± 7.55^∗#^	15.84 ± 8.18	−9.31 ± 6.41	−4.87 ± 7.11	−8.57 ± 11.28
SRS	12.16 ± 3.17^∗#^	7.45 ± 2.69^∗#^	5.79 ± 2.79	−1.16 ± 3.21	−3.95 ± 3.67	0.65 ± 3.09
HRS	1.14 ± 11.22	−11.42 ± 9.28	15.72 ± 13.74	−9.29 ± 6.06	−12.66 ± 9.60	0.23 ± 9.01
RP	3.14 ± 2.82	4.21 ± 3.32	5.74 ± 3.22^#^	−7.08 ± 4.95	0.13 ± 5.41	−10.37 ± 5.58
MDA	14.07 ± 3.21^∗^	14.70 ± 4.30^∗^	21.79 ± 3.59^∗^	15.83 ± 3.81^∗^	14.55 ± 3.71^∗^	23.65 ± 4.87^∗^

PC: protein carbonyls; TBARS: thiobarbituric acid reactive substances (malondialdehyde measured spectrophotometrically); TAC: total antioxidant capacity; GSH: reduced glutathione; CAT: catalase; sORP: static oxidation-reduction potential; cORP: capacity oxidation-reduction potential; SRS: superoxide radical scavenging; HRS: hydroxyl radical scavenging; RP: reducing power; MDA: malondialdehyde (measured by HPLC-DAD). Values are expressed as mean ± SEM. ^∗^Statistically significant compared with preexercise values (*p* < 0.05). ^#^Statistically significant between trained and untrained at the same time point.

**Table 4:** Correlation analysis between superoxide radical-scavenging (SRS) activity, hydroxyl radical-scavenging (HRS) activity, reducing power (RP), and total antioxidant capacity (TAC).

	24 h			48 h			72 h		
	HRS	SRS	RP	HRS	SRS	RP	HRS	SRS	RP
*Trained*									
HRS		−0.153	0.514^∗^		−0.081	0.253		0.155	0.336
SRS			−0.107			0.137			−0.068
TAC	0.178	−0.126	0.061	−0.248	0.008	−0.214	−0.137	0.077	−0.327
*Untrained*									
HRS		0.352	−0.117		−0.385	0.166		−0.412	−0.328
SRS			−0.127			−0.069			0.130
TAC	0.230	0.325	−0.199	−0.007	−0.117	0.346	0.356	−0.069	−0.123

^∗^Significant correlation (*p* < 0.05).

## Data Availability

All data, tables, and figures in this manuscript are original and are available upon request.
